# Clinical application of gelatin sponge microparticles combined with pirarubicin for hepatic transcatheter arterial chemoembolization in breast cancer liver metastasis treatment: results of a single-center long-term study

**DOI:** 10.1186/s12957-021-02332-0

**Published:** 2021-08-21

**Authors:** Guang Sheng Zhao, Song Liu, Ying Liu, Jian Ma, Ruo Yu Wang, Jie Bian, Jun Zhou, Jian Lin Wu, Yue Wei Zhang

**Affiliations:** 1 Interventional Medicine Center, Affiliated Zhongshan Hospital of Dalian University, No. 6 Jie Fang Street, Dalian, 116001 Liaoning Province China; 2 Interventional Medicine Center, Linyi Cancer Hospital, 6 East Lingyuan Street, Linyi, 276001 Shandong Province China; 3 Hepatobiliary and Pancreatic Center, Beijing Tsinghua Changgung Hospital, 168 Litang Road, Beijing, 102218 Changping District China; 4 Cancer Treatment Center, Affiliated Zhongshan Hospital of Dalian University, No. 6 Jiefang Street, Dalian, 116001 Liaoning Province China; 5 Medical Imaging Center, The Second Hospital of Dalian Medical University, No. 467 Zhongshan Raod, Shahekou District, Dalian, 116044 Liaoning Province China; 6 Medical Imaging Center, Affiliated Zhongshan Hospital of Dalian University, No. 6 Jie Fang Street, Dalian, 116001 Liaoning Province China

**Keywords:** Breast tumor, Liver metastasis, TACE, Gelatin sponge microparticles, Efficacy

## Abstract

**Objective:**

To retrospectively analyze the safety and long-term clinical efficacy of gelatin sponge microparticles combined with the chemotherapy drug pirarubicin for hepatic transcatheter arterial chemoembolization (GSMs-TACE) in order to treat breast cancer liver metastasis (BCLM).

**Methods:**

Twenty-seven BCLM patients who underwent GSMs-TACE from July 2010 to July 2016 were enrolled. Tumor target blood vessels were slowly and regionally embolized with absorbable gelatin sponge particles and pirarubicin injections. Plain computed tomography (CT) scans and biochemical indexes were re-examined at 4 days after treatment, and enhanced CT scans or magnetic resonance images and biochemical indexes, 1 month later. For patients with stable tumors, the follow-up period was 2 to 3 months, and the tumor response was evaluated using Modified Response Evaluation Criteria in Solid Tumors. Adverse reactions, survival time, and prognostic factors were assessed.

**Results:**

By October 2019, 27 patients with BCLM had undergone GSMs-TACE, with an average of 2.44 ± 1.58 treatments. The 1-, 3-, and 5-year survival rates were 62.96%, 22.22%, and 14.81%, respectively, and the mOS was 22.0 months. No serious complications, such as acute liver failure and liver abscess, had occurred. There were two cases of acute cholecystitis that recovered after symptomatic treatment. Multivariate analysis of the prognosis showed that the primary tumor size, number of metastatic lymph nodes, estrogen receptor/progesterone receptor (ER/PR) status, and time to postoperative liver metastasis and combination therapy were statistically significant.

**Conclusions:**

The overall prognosis of BCLM was poor. GSMs-TACE was safe and effective for BCLM treatment and could prolong the median survival time of patients. Therefore, it is worthy of widespread clinical application.

## Introduction

Breast cancer is the most common type of malignant tumor in women, with ~ 50% of patients experiencing local recurrence or distant metastasis after surgical resection. Approximately, 50% of these patients have liver metastasis, and 5–12% of these patients relapse after surgical resection and systemic chemotherapy [1]. The prognosis of breast cancer liver metastasis (BCLM) is very poor (median overall survival (mOS), 18–24 months), and it is even worse in patients in whom chemotherapy fails [2, 3]. As a means of local treatment, the mOS can reach up to 47 months after transcatheter arterial chemoembolization (TACE) for BCLM [4]. Gelatin sponge microparticles for hepatic transcatheter arterial chemoembolization (GSMs-TACE) is a TACE technique involving the use of absorbable gelatin sponge particles. Previous studies have shown the efficacy and safety of GSMs-TACE in the treatment of primary liver cancer and liver metastasis of colorectal cancer [5–7]. In this study, we retrospectively analyzed the feasibility and long-term clinical efficacy of GSMs-TACE in 27 BCLM patients.

## Materials and methods

### Case selection

The inclusion criteria were as follows: (i) younger than 80 years of age; (ii) confirmed diagnosis of BCLM; (iii) failure of first-line chemotherapy, inability to undergo surgical resection, or refusal to undergo surgery; (iv) liver function classified as Child Pugh A-B and a liver tumor volume less than two-thirds of the total liver volume; (v) previous disease progression after chemotherapy, intolerance to current chemotherapy, or refusal of systemic chemotherapy; (vi) expected survival period of more than 3 months; and (vii) having signed the informed consent form and agreed in writing to voluntarily participate in this study.

The exclusion criteria were as follows: (i) allergy to contrast agents or chemotherapy drugs; (ii) significant heart, kidney, or brain dysfunction or liver function classified as Child Pugh C; and (iii) physical strength (PS) score greater than 2.

### GSMs-TACE technique

The Seldinger technique was used to routinely puncture the right femoral artery and introduce a RH catheter (5F-RH catheter, Terumo Corp., Japan) for angiography of the coeliac trunk and common hepatic artery. Using preoperative imaging data, we interpreted the DSA imaging results and conducted supplementary angiography of the diaphragmatic artery, superior mesenteric artery, left gastric artery, and other collateral vessels, as necessary, to identify the vessels supplying blood to the tumor. All patients underwent intensive CT and other examinations before surgery to assess the possible tumor supply blood vessels. GSMs (Hanzhou Alicon Pharmaceutical Technology Co., Ltd., China) with different diameters and inner diameters were selected according to the imaging manifestations, with two specifications mainly applied, 150–350 μm and 350–560 μm. A 30–50-mg pirarubicin (Zhejiang Hisun Pharmaceutical Co., Ltd., China) injection was administered. The chemotherapy drug was diluted to approximately 30 ml with 0.9% NaCl, mixed with GSMs to prepare a microparticle suspension, and slowly injected into the tumor blood supply arteries. The criteria suggesting an embolism were as follows: (i) stagnated blood flow in the blood supply vessels of the tumor; and (ii) disappearance of tumor staining in angiography. Routine administration was required after treatment, and acid inhibition, liver protectant, fluid replacement, and symptomatic treatments were administered for 3 to 5 days.

### Efficacy evaluation and adverse reaction observation

Non-enhanced computed tomography (CT) scanning was performed at 4 days after the procedure, and then at 1 month, we performed either contrast enhanced CT scanning or magnetic resonance imaging (MRI) in order to assess the size and necrosis within the embolized lesions. CT image processing software was used to estimate the volume of the liver and mass. The biochemical indexes were re-examined at 4 days, 7 days, and 1 month after treatment. Patients in complete response (CR) underwent abdominal enhanced CT/MRI scans at intervals of 2 to 3 months. mRECIST was applied to evaluate the clinical efficacy, observe for tumor progression, comprehensively evaluate the effects of the intervention, and determine whether the patient would need to receive the interventional treatment again. Adverse reactions were observed during the treatment, for which treatments were administered and records were actively updated.

### Analysis of survival time and prognostic factors

The survival rates of patients after interventional treatment and the overall survival of patients after liver metastasis were observed and summarized. For the patients who refused interventional treatment or another comprehensive treatment, continuous clinical follow-up was carried out. The cut-off time for follow-up was the death of the patient or the end of the follow-up period. Multivariate analysis of prognosis was carried out after the cut-off date of the follow-up period.

### Statistical methods

SPSS 20.0 software was used to analyze the data. Measurement data were expressed as the mean ± standard deviation $(\overline{x }\pm s)$. The *t* test was used for intergroup comparisons of normally distributed data, and the rank-sum test was used for intergroup comparisons of non-normally distributed data. The *r* test was used for intergroup comparisons of enumeration data, the Kaplan–Meier statistical method was used to calculate the survival rate, the log-rank test was used for univariate analysis, and the Cox model was used for multivariate analysis. *P* < 0.05 was taken as the level of statistical significance.

## Results

### Patient information

From July 2010 to July 2016, 27 patients with BCLM continuously received GSMs-TACE. All patients were females, with ages ranging from 33 to 79 years, and an average age of 55.17 ± 9.06 years. Sixteen BCLM patients were diagnosed with liver metastasis within 24 months. The time to liver metastasis was 8–96 months (average time, 30.88 months) after surgical resection. All patients were fully informed of the study procedure and signed an informed consent form before surgery. The average number of interventional treatment sessions for the 27 BCLM patients was one to six sessions, with an average of 2.44 ± 1.58 sessions (Table 1).

**Table 1:** Baseline characteristics of 27 patients with BCLM

Clinical features	Value
Average age (years) (range)	55.17 ± 9.06 (33–79)
60 years	21
≥ 60 years	6
Menstrual state	
Premenopausal	9
Postmenopausal	18
Pathological subtype	
Invasive ductal carcinoma	26
Invasive lobular carcinoma	1
Primary tumor size (cm)	
2	15
2–5	6
≥ 5	5
Invasion of chest wall skin	1
Axillary metastatic lymph nodes (number)	
0	14
1–34	8
≥ 4	5
ER/PR	
+	18
−	9
Her-2	
+	15
−	12
Time to postoperative liver metastasis (months)	
< 24	16
≥ 24	11
Intrahepatic lesions (number)	
Single	5
Multiple	22
Intrahepatic lesion size (cm)	
3	3
≥ 3	24
Whether combination therapy was adopted	
Yes	18
No	9
Whether complicated by extrahepatic metastasis	
Yes	16
No	11
Number of interventional treatment sessions (range)	2.44 ± 1.58 (1–6)

*ER/PR* estrogen receptor/progesterone receptor

### Tumor response after interventional treatment

**Fig. 1:**
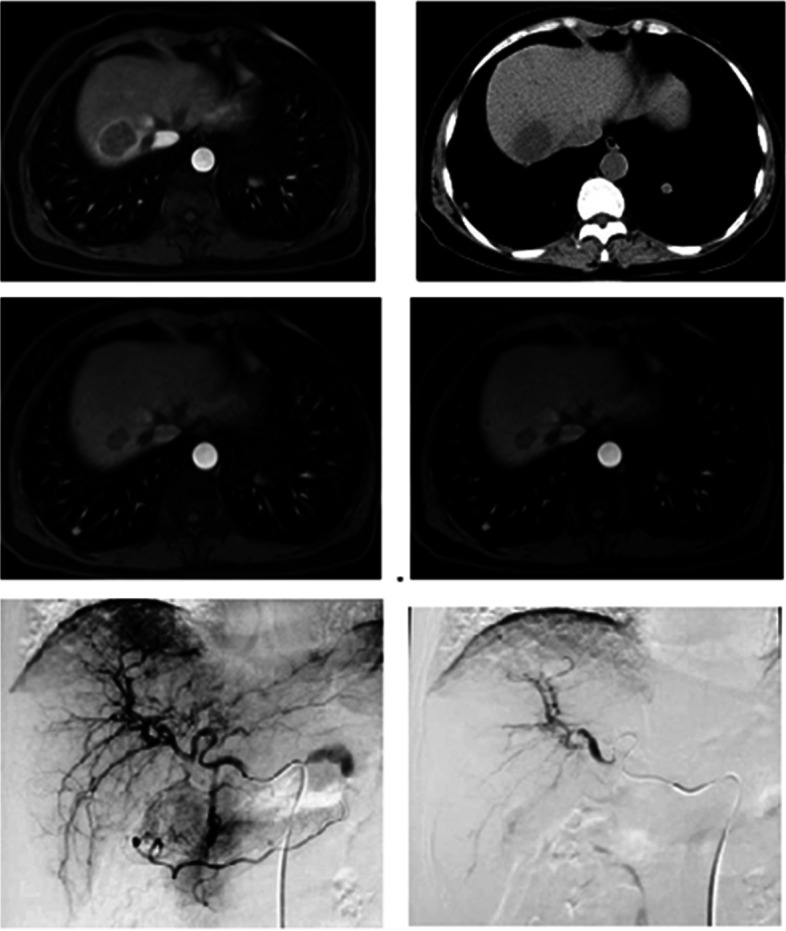
**a** Enhanced MRI images before interventional treatment. **b** Plain CT scan showed significant low-density changes of the tumor 4 days after treatment. **c** Enhanced MRI showed that the lesion was significantly reduced, and no significant enhancement was found 3 months after treatment. **d** Twelve months after treatment, the features of the lesion were basically the same as those in **c**, without significant changes. **e** Imaging during the interventional treatment showed nodular tumor staining near the diaphragm apex, which was supplied by branches of the hepatic artery. **f** Tumor staining had completely disappeared in the image after treatment

Four days after the interventional treatment, non-enhanced CT (NECT) scanning of the abdomen revealed that the tumors showed a uniform distribution, with significant low-density necrotic changes. Cellular necrotic changes were observed in some cases. In this study, mRECIST was used to evaluate the tumor response after interventional treatment. One month after the interventional treatment, the CR and objective response rate (ORR) were 25.93% and 74.07%, respectively, and the disease control rate (DCR) reached 96.30%. The interventional treatment scheme and imaging follow-up of typical cases are shown in Fig. 1 (Table 2).

**Table 2:** Tumor response after GSMs-TACE treatment (*n*, %)

Post-operation	*n*	CR (%)	PR (%)	SD (%)	PD (%)	ORR (%)	DCR (%)
1 month	27	7 (25.93)	13 (48.15)	6 (22.22)	1 (3.70)	20 (74.07)	26 (96.30)

### Survival time and survival curve

**Fig. 2:**
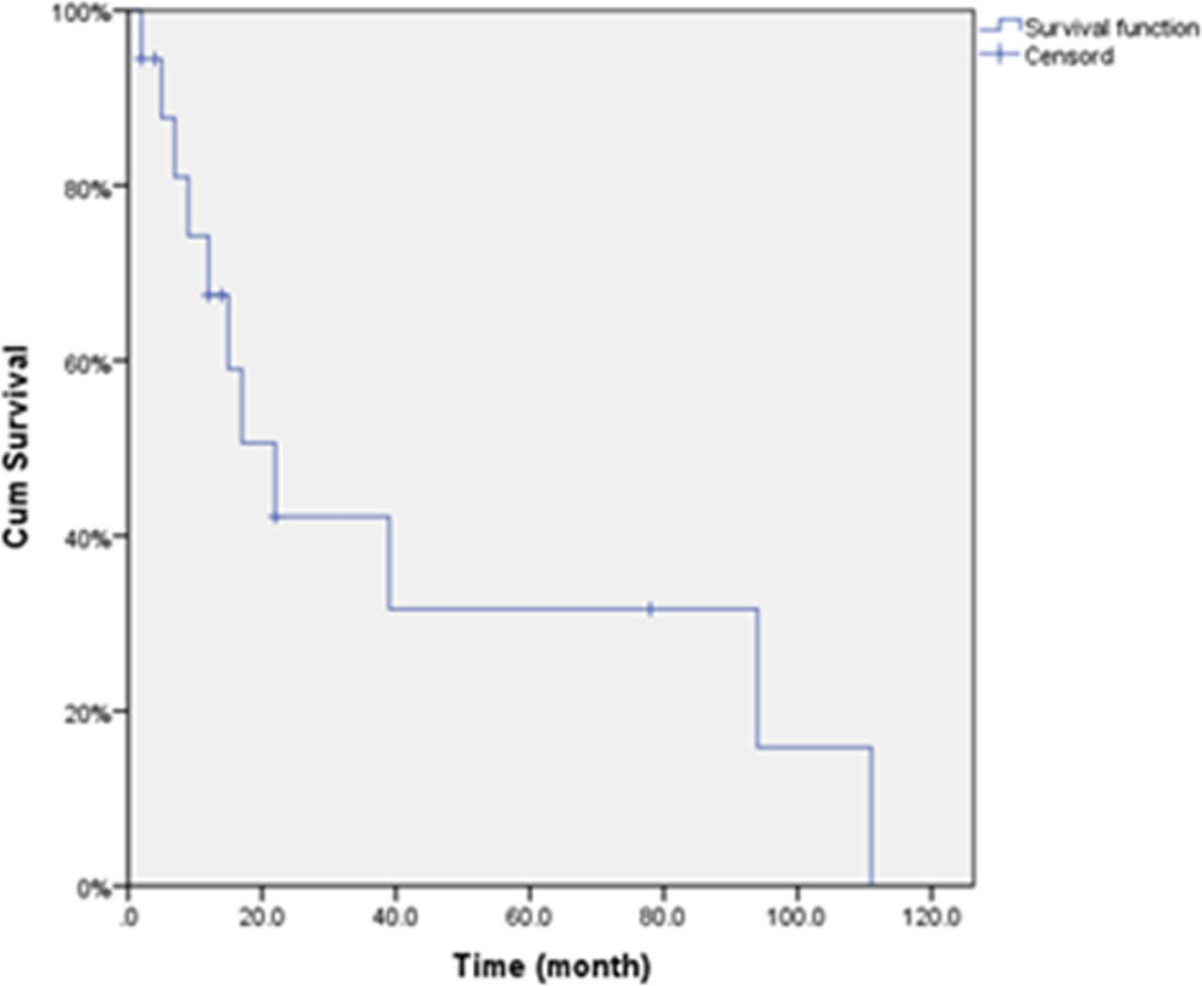
Survival curve of BCLM after GSMs-TACE, with an mOS of 22 months

In October 2019, the follow-up period was 2–111 months (average time, 26.06 months). The 1-, 3-, and 5-year survival rates were 62.96%, 22.22%, and 14.81%, respectively, and the mOS was 22 months. The mOS after liver metastasis was 23 months (Table 3 and Fig. 2).

**Table 3:** Survival time

1-year survival rate		3-year survival rate		5-year survival rate		mOS	mOS after metastasis
N	%	N	%	N	%		
17	62.96	6	22.22	4	14.81	22.0 (95% CI 10.789–33.211)	23.0 (95% CI 16.443–29.557)

### Adverse reactions and complications

After treatment, 21 patients developed fever, which was considered to be related to tumor necrosis and absorption, and it subsided within 14 days. Upper abdominal pain was reported in 16 cases (59.26%), and nausea and vomiting, in 10 cases (37.04%). The reactions were mild and usually relieved within 24 h. All patients had transient liver function damage, and they recovered 7–10 days after liver protection treatment, without any statistically significant difference compared with their preoperative conditions. There were two cases of acute cholecystitis due to possible non-target embolization, which was relieved by symptomatic treatment. No serious complications were observed in the study group (Tables 4 and 5).

**Table 4:** Change of liver function before and after GSMs-TACE $(\overline{x}\pm s)$

	Pre-TACE	7 days after TACE	*P*
ALT (U/L)	50.70 ± 16.15	59.33 ± 17.53	0.052
AST (U/L)	49.41 ± 14.27	57.79 ± 16.91	0.054
TBIL (μmol/L)	24.53 ± 10.13	30.63 ± 11.58	0.063
ALB (g/L)	36.04 ± 7.23	32.19 ± 4.06	0.051

**Table 5:** Adverse events after GSMs-TACE treatment (*n* = 27)

**Adverse events**	Level 1	Level 2	Level 3	Level 4
Fever	15	5	1	0
Pain	13	2	1	0
Nausea and vomiting	7	2	1	0
Abscess	0	0	0	0
Cholecystitis	2	0	0	0
Pancreatitis	0	0	0	0
Renal insufficiency	1	0	0	0
Hepatic insufficiency	20	6	1	0
Bone marrow arrest	2	1	0	0

### Analysis of prognostic factors

Eleven indexes, including age, menstrual status, primary tumor size, number of metastatic lymph nodes, estrogen receptor/progesterone receptor (ER/PR) status, and number and size of liver metastases, were included in the prognostic factor analysis. Five of these indexes were statistically significant according to multivariate analysis, including primary tumor size, number of metastatic lymph nodes, ER/PR status, time to postoperative liver metastasis, and combination therapy (Table 6).

**Table 6:** Analysis of the prognostic factors

Factor	*N*	Single factor			Multiple factors		
		RR	95% CI	*P*	RR	95% CI	*P*
Age							
< 60 years	21	1			1		
≥ 60 years	6	1.58	0.957–2.164	0.086	1.97	1.180–2.668	0.059
Menstrual status							
Premenopausal	9						
Postmenopausal	18	1.792	1.057–2.601	0.034	1.32	0.847–1.809	0.072
Primary tumor size (cm)							
< 2	15	1			1		
2–5	6	1.675	1.011–2.917	0.045	1.115	0.980–3.028	0.021
≥ 5	5	2.174	1.039–4.632	0.037	2.538	1.157–4.251	0.001
Tumor involving chest wall	1	3.548	1.387–8.171	0.040	1.478	1.245–1.773	0.004
Number of axillary metastatic lymph nodes							
0	14	1			1		
1–3	8	1.726	0.962–2.928	0.049	1.971	1.939–3.752	0.041
≥ 4	5	2.279	1.062–3.373	0.001	1.960	1.017–3.758	0.037
ER/PR status							
Positive	18	1			1		
Negative	9	2.120	1.204–3.243	0.024	2.310	1.673–3.554	0.000
Her-2 status							
Positive	15	1			1		
Negative	12	1.490	1.245–1.783	0.01	1.971	1.193–3.255	0.150
Time to postoperative liver metastasis (months)		1			1		
24	16						
≥ 24	11	1.971	1.073–3.578	0.028	0.561	0.327–0.965	0.037
Number of intrahepatic lesions (*N*)							
Single	5	1			1		
Multiple	22	1.630	1.074–3.383	0.056	1.67	1.243–2.255	0.079
Size of intrahepatic lesions (cm)							
< 3	3	1			1		
≥ 3	24	1.524	0.448–2.171	0.012	1.822	1.028–3.339	0.060
Presence of extrahepatic metastasis							
Yes	16	1			1		
No	11	1.578	1.012–3.654	0.001	0.258	0.214–1.012	0.593
Was combination therapy adopted?							
Yes	18	1			1		
No	9	2.275	1.462–3.540	0.000	0.891	0.799–0.993	0.037

## Discussion

TACE has several advantages over systemic chemotherapy [8]. First, this method involves the arterial blood supply of the tumor. It delivers higher concentrations of the chemotherapy drug to tumor cells, rather than all organs of the body, thus eliminating damage caused to them. Second, alongside chemotherapy drugs, TACE can embolize tumor supply blood vessels, rapidly causing tumor necrosis or apoptosis because of the disappearance of supporting blood vessels. This process takes less time compared with other angiogenesis inhibitors such as targeted drugs. However, tumor blood vessels are the basis of TACE in the treatment of BCLM. In metastatic liver cancer, because of the small blood vessels, tumors usually show a lack of blood supply and poor deposition of iodized oil, which are considered the main causes for the poor TACE results [9].

In recent years, microparticle-TACE therapy for primary liver cancer and metastatic liver cancer has further improved the tumor response and control rate [9–11]. Our previous research results also confirmed the feasibility of GSMs-TACE in the treatment of metastatic liver cancer [5, 12]. The results of this study showed that the disease control rate (DCR) of GSMs-TACE treatment for BCLM at 1 month after surgery was 96.30%, and the mOS reached 22 months. Lin et al. reported that when treating BCLM with doxorubicin-loaded microparticles, the mOS reached 17 months, significantly better than that with local radiotherapy [11]. The two points above are considered the main reasons for the effectiveness of this treatment. Absorbable gelatin sponge particles, which are medium-term embolic agents, generally degrade in about 2 weeks. GSMs-TACE can be used for the regional arterial embolization of tumors, which is distinct from the utilization of other types of permanent embolic particles and is equivalent to surgical resection and ablation, as the periphery of the tumor has more activity and is a common site for tumor recurrence [13]. Gelatin sponge particles have a specific drug-loading limit [14], and the slow release of chemotherapy drugs can further improve the tumor response rate. In terms of postoperative adverse events, this study shows that postoperative complications of GSMs-TACE were mainly of grades I or II. Compared with that of other methods, the number of interventional treatment sessions was also significantly reduced, with more protection to liver and kidney functions. Gelatin sponge particles, as absorbable embolic particles, had no occupying effect in the liver compared with iodized oil, which is conducive to the evaluation of the efficacy after TACE.

This study shows that the mOS after GSMs-TACE was 22.0 months, and the 5-year survival rate was 14.81%. Compared with the mOS in patients with a similar baseline status and in whom other types of TACE methods were used, the treatment results were still satisfactory. Univariate analysis of the prognosis in this study also shows that the prognosis of GSMs-TACE may be better for single tumors and BCLM with tumors smaller than 3 cm or without extrahepatic metastasis, and combined treatment will further improve the prognosis. Therefore, we hold the opinion that GSMs-TACE and other local treatment methods combined with systemic treatment may be more suitable for BCLM, especially for BCLM with lesions less than 3 cm or 5 cm without extrahepatic metastasis. However, this process may require multidisciplinary consultation for successful completion [15–17], and the study of the mutant circular RNA map also makes treatment of BCLM possible [17].

In summary, GSMs-TACE is feasible for the treatment of BCLM, especially for patients with advanced BCLM who are unsuitable candidates for chemoradiotherapy and surgical resection. This study is a single-center retrospective study with selection bias, and its results are also affected by the small sample size. Therefore, a prospective clinical study with a larger sample size is necessary.

## Data Availability

All data generated or analyzed during this study are included in this published article.
